# Infected grasses as inoculum for *Fusarium* infestation and mycotoxin accumulation in wheat with and without irrigation

**DOI:** 10.1007/s12550-022-00470-2

**Published:** 2022-10-25

**Authors:** Marina Gerling, Laura Petry, Dietmar Barkusky, Carmen Büttner, Marina E. H. Müller

**Affiliations:** 1 Leibniz Centre for Agricultural Landscape Research (ZALF), Eberswalder Str. 84, 15374 Müncheberg, Germany; 2 Albrecht Daniel Thaer-Institute, Faculty of Life Science, Department of Phytomedicine, Humboldt-Universität Zu Berlin, 14195 Berlin, Germany

**Keywords:** Grasses, *Fusarium*, Mycotoxins, Reservoir, Irrigation, Non-host plants

## Abstract

Grasses growing next to agricultural fields influence the *Fusarium* abundance, the species composition, and the mycotoxin accumulation of wheat plants, especially the field parts directly adjacent to grasses, are highly affected. Grasses are a more attractive and suitable habitat for *Fusarium* fungi compared to other arable weeds and occur at mostly every semi-natural landscape element (e.g., kettle holes, hedgerows, field-to-field-borders). In our study, we analyzed the ability of a highly *Fusarium* infected grass stripe (*F. graminearum*, *F. culmorum*, *F. sporotrichioides*) to infect an adjacent wheat field with these species. Results show that the primary inoculated *Fusarium* species were as well the dominant species isolated from the wheat field. Regarding transects originating from the grass stripe going into the field, the results demonstrate that wheat ears next to the infected grass stripe have a higher *Fusarium* abundance and furthermore show higher mycotoxin accumulation in the wheat kernels. This effect was highly promoted by irrigation. Therefore, grass stripes next to arable fields must be considered as reservoirs for fungal infections and as a source for a contamination with mycotoxins.

**Supplementary Information:**

The online version contains supplementary material available at 10.1007/s12550-022-00470-2.

## Introduction

Wheat (*Triticum aestivum* L.) is one of the most economically important crop on which global food security is depending. Unfavorably, numerous plant diseases can reduce yields and thereby threaten the food security. Wheat is highly susceptible to fungal infections and fungal associated diseases can be responsible for 15–20% of crop losses worldwide each year (Figueroa et al. 2018; Savary et al. 2019), whereas viral and bacterial diseases are usually less important (Oerke 2006).

Regarding fungal infections like wilts, rots, and blights (Rampersad 2020), mainly *Fusarium* head blight (FHB), the economically most devastating wheat disease, caused by state of the art up to 19 different *Fusarium* species, poses farmers to challenges (Parry et al. 1993; Champeil et al. 2004; Landschoot et al. 2011; Müller et al. 2012). Growth of fungal pathogens in wheat as well as the mycotoxin is highly influenced by agricultural, topographic, and also climatic factors (Dill-Macky and Jones 2000; Vogelgsang et al. 2019). A strong multifactorial influence of tillage, previous crop, variety, soil and air moisture, and other site factors on fungal pathogen community and on mycotoxin accumulation in wheat is observed. Different cropping systems and the interaction between different phytopathogenic fungi and bacteria also determine the incidence and impact of fungal plant diseases (Classen et al. 2005; Drakopoulos et al. 2021). Mycotoxins produced by *Fusarium* fungi during its growth in the wheat ears could have different toxic effects on both animal and human health after uptake of contaminated wheat products (Zain 2011; Chhaya et al. 2021). Deoxynivalenol (DON), zearalenone (ZEN), and nivalenol (NIV) are the most frequently reported mycotoxins and mostly detected on wheat, maize, rice, barley, and rye (Visconti et al. 1992; Bottalico 1998). In Europe, DON and ZEN, mainly produced by *F. graminearum* and *F. culmorum* are the most detected toxic secondary metabolisms. *F. graminearum* is more common in warmer, while *F. culmorum* is more often to be found in colder regions of Europe (Pasquali et al. 2016).

However, crop residues enduring in the field from last season are regarded as the primary inoculum for an infection with FHB (Landschoot et al. 2011; Leplat et al. 2013). In recent years, also (arable), weeds are of interest as reservoir for fungal spores and as a source infection for crop plants. Weeds are part of every agricultural system and occur alongside the crops in the field season, more since a lot are resistant against herbicides. Seventy percent of gramineous weed species in China were already resistant to herbicides, making them hard to control (Dong et al. 2020). At semi-natural habitats (e.g., hedgerows in the field, field margins, kettle holes), weeds can even grow permanently over several field seasons because they were not harvested alongside the crops, giving species of *Fusarium* a suitable habitat to overwinter or outlast times when their main host is not available. Originating from these weeds, the fungal spores can immigrate into the field by wind, rain and through transport by organisms such as insects (Rossi et al. 2002; Paul et al. 2004; Heitmann et al. 2021; Hoffmann et al. 2021). However, weeds offer broad host range for fungal pathogens, including *Fusarium*, and can act as both alternative and alternate host for overwintering (Kumar et al. 2021), development and sporulation. Especially gramineous weeds harbor high abundances of *Fusarium* fungi while are also infested with a diverse species composition (Lofgren et al. 2018; Fulcher et al. 2019a, b B; Dong et al. 2020; Gerling et al. 2022). Wild grasses were also determined to be important in the survival of *F. graminearum*, *F. sporotrichioides*, *F. equiseti*, *F. avenaceum*, *F. poae*, *F. oxysporum*, and *F. culmorum* (Landschoot et al. 2011). Senescent but also fresh weeds were analyzed in Croatia, isolating different *Fusarium* species with *F. graminearum* as the most frequent one (Postic et al. 2012). Also, Mourelos et al. (2014) isolated *F. graminearum* from a great variation of weed species, and Suproniene et al. (2019) detected that the *Fusarium* strains isolated from weeds are also pathogenic for wheat. This leads to the assumption that also weeds, besides crop residues and soil, are an important habitat for fungal pathogens to survive and sporulate. The community composition of *Fusarium* on arable weeds could be depending on many factors including the study side, crops, the weeds, and the climate condition. Regional differences in species profile may exist due to different crop rotation systems and local climatic conditions (especially temperature and precipitation) (Liu and Van Der Fels-Klerx 2021).

Organic farming is becoming increasingly important in European agriculture (Hansen et al. 2001). As a result, the control of weeds with herbicides is severely limited and weeds are becoming more common in crop fields. Also, the establishment of flower stripes or semi-natural landscape structures such as hedges and wide edge grass stripes to preserve biodiversity (pollinators, amphibians, ground beetles, birds) (Benton et al. 2003; Lozada-Gobilard et al. 2021) cause weeds and grasses to be more and more common in agricultural landscapes and thus — besides all positive effects — also a habitat for phytopathogenic mycotoxin producing fungi. The impact of these measures on the spread of plant diseases in adjacent cereal fields is still unknown.

The life cycle of filamentous fungi like *Fusarium* spp. is highly influenced by moisture conditions in their living environment. Various studies mentioned environmental conditions, including relative humidity, as essential for the development of *Fusarium* (Backhouse et al. 2004; Osborne and Stein 2007; Landschoot et al. 2011; Kelly et al. 2015). Parikka et al. (2012) expected a shift in the species composition of *Fusarium* on cereal grain in northern Europe due to changing climate conditions. Liu and Van der Fels-Klerx (2021) mentioned that the increase of precipitation but also increasing temperatures in Europe may promote the spread of *Fusarium* spp. The changing weather conditions caused by climate change do not only influence the abundance of *Fusarium*, but also the presence of mycotoxins in wheat crops among others, due to their existence depending on multiple factors including temperature and precipitation. However, the contribution of weeds in the *Fusarium* head blight disease cycle is not fully understood by now (Keller et al. 2014; Miedaner et al. 2017; Fulcher et al. 2019a, b; Martínez et al. 2021).

Therefore, we hypothesize that (1) a highly *Fusarium* infected grass stripe acts as an infection source of different *Fusarium* species for the adjacent wheat field, with this effect decreasing with increasing distance from the grass stripe; (2) the mycotoxins produced by the *Fusarium* are accumulated in high concentrations in this transition zone from the grass stripe into the field and thus differ from the average toxin concentration inside the field; (3) these transmission effects are enhanced by irrigation on one half of the field and lead to higher *Fusarium* infestation and mycotoxin concentrations of wheat plants in this field side.

## Material and methods

In 2020, a field experiment was conducted in a winter wheat field on the research area at the Leibniz Centre of Agricultural Landscape Research (ZALF) Müncheberg to evaluate the influence of highly infected grasses and irrigation on the spread and the diversity of *Fusarium* fungi.

### Study site

The examined winter wheat field (preceding crop: winter wheat), located in Müncheberg, Germany (52.5176 N; 14.1217 E), was 3339 m^2^ in total (53 m wide × 63 m length), divided into two field sides. The soil type was Sl4D soil (sandy clay loam pallid soil). The wheat variety “Tobak” was cultivated, which is susceptible for Fusarium head blight (susceptibility of the wheat cultivar to an infection with *Fusarium* spp. expressed as a rating of 7 on a scale from 1 to 9; Anonymous, 2022). Detailed information on the agricultural management practices and applications are shown in Table S1. All the residues of the previous crop were removed from the field; the soil was ploughed and then cleared again. Emerging weeds in the field were suppressed or minimized at all times with herbicides or by hand. No fungicides against *Fusarium* infections were applied. Additionally, herbicides decimate the weeds emerging in winter. In spring, the field was checked again, and the few remaining weeds were eliminated. The management of the trial aimed very consistently at minimizing all sources of infection except the inoculated grass stripe.

In October 20, 2020, we implemented a grass stripe (*Lolium multiflorum* L. (85%), *Lolium* × *hybridum* Hausskn. (15%)) with an area of 159m^2^ (53 m wide × 3 m length). All other grass stripes along the field were mowed throughout the experimental period. One wheat field side was irrigated (5–10 mm/m^2^ per week) from the end of March 24, 2021, until the end of the vegetation period (harvest: 2 August 2021) using circular sprinklers (Perrot ZE30, Perrot Regnerbau Calw GmbH, Althengstett, Germany).

### Inoculation of the grass stripe

The grass stripe was infected with *Fusarium* fungi through soil inoculation. *Fusarium* isolates used as inoculum in this study originated from wheat plants and maintain in the culture collection of fungi of the working group “Fungal Interactions” at the ZALF in Müncheberg. To perform the inoculation, wheat kernels were infected with three different *Fusarium* species: *F. graminearum*, F*. culmorum*, and *F. sporotrichioides*. Wheat kernels were moistened with sterile water and autoclaved three times, before infected with *Fusarium* fungi by mycelium-covered nutrient media (potato dextrose agar PDA, Merck, Heidelberg, Germany) and incubated by 24 °C for 14 days in the dark. In total, 3600-g wheat kernels were used, 1200 g per *Fusarium* species. Inoculum for every *Fusarium* species was produced separately. Afterwards, the infected kernels from each species were uniformly spread over the soil (7.5-g inoculum from each species/m^2^) in November 24, 2020, and lightly pressed into the ground. *F. graminearum* was chosen for being the main causal agent of FHB in wheat and other small-grain cereals in Europe. *F. culmorum* is a soil-borne pathogen and also highly associated with FHB. *F. sporotrichioides* is less pathogenic to wheat, though was also selected due to its high abundance on grasses at small water bodies on wheat fields in Brandenburg/Germany (Gerling et al. 2022).

### Microclimate data

Soil humidity (m^3^/m^3^), air humidity (%), leaf wetness (%), and air temperature (°C) were monitored from March 2021 through the duration of the experiment using 6 microclimatic measuring stations (Hobo H21 Micro Station, Onset Computer Corporation, MA, USA) with soil moisture smart sensor (S-SMD-M005), leaf wetness smart sensor (S-LWA-M003), and temperature/RH smart sensor (S-THB-M002) all of them provided by Onset Computer Corporation (Bourne, MA, USA). Except for the soil humidity sensor (measurement in 10 cm depth), all sensors were positioned at a height of 30 cm above ground. The sensors provided measurements every hour.

### Sampling design

Samples of the grasses from the inoculated grass stripe were taken in May and June 2021 at 6 different sampling points. Fifteen grass blades were cut 2 cm above the ground. The abundance of *Fusarium* and furthermore the species composition were analyzed by culture-dependent methods to monitor the inoculation success and the development of the inoculated species. Grass samples in June 2021 were taken while wheat was flowering and was meanwhile most susceptible to fungal infections (Góral et al. 2019).

In July 2021, 2 weeks after full flowering at BBCH stage of medium to late milk development (BBCH 75–77) (Zadoks et al. 1974), wheat ears were sampled a long six transects starting next to the grass stripe up to 64 m into the wheat field. Sampling points were set at 1 m, 2 m, 4 m, 8 m, 16 m, 33 m, and 64 m. The transects were 5 m apart from each other. Fifteen different wheat ears were randomly picked in a 0.5 m area around the sampling point and cut 2 cm below the ear. Wheat ears were sampled for further investigations regarding the abundance of filamentous fungi and the diversity of *Fusarium* species. All samples were collected in paper bags and immediately transported to the laboratory for further investigations. Collected plants were analyzed by culture-dependent and culture-independent methods for the presence of fungi, mainly filamentous fungi of the genera *Fusarium*.

### Laboratory analyses

#### Culture-dependent method

Potato dextrose agar (PDA; Merck, Heidelberg, Germany) supplemented with chloramphenicol to suppress bacterial growth, and synthetic nutrient agar (SNA) (Nierenberg 1976) were used as described detailed by Leslie and Summerell (2006) to determine the colony forming units (cfu) per gram of fresh matter of *Fusarium* (FUS_cfu/gFM) and the species analysis of *Fusarium*. Regarding the grasses, ten pieces (about 1 cm length) of each grass sample were cut out and weighted (Kern 572–35; Kern&Sohn GmbH Balingen-Frommern, Germany) to calculate the colony forming units to 1 g of plant fresh matter for further statistical analyses. Afterwards, grass pieces were plated onto two PDA containing petri dishes (diameter 9 cm); five pieces (mixed from leaves, stems, and inflorescences) were placed on each plate.

Wheat samples were prepared as described above by using 10 kernels per sampling point from 10 different wheat ears (one kernel per ear), randomly chosen from the bottom, the middle and the tip of the ear.

Plated samples, both grass samples and wheat samples, were incubated for 2 days at 24 °C in darkness to support the fungal growth and further 2 days under UV light (12 h UV light/12 h daylight) at room temperature to support sporulation and coloration of the mycelium. After 4 days, the colonies of *Fusarium* (FUS_cfu/gFM) were counted on each petri dish.

For the morphological identification of different *Fusarium* species, again PDA and SNA petri dishes were used. Colonies from the initial plate were transferred onto one plate of both PDA and SNA. New petri dishes were treated as described above, but with of a longer UV light treatment depending on the growth rate of the different *Fusarium* species. UV light treatment up to 14 day for slow growing species is possible. The cultures grown on PDA supports the formation of the typical coloring of the fungal mycelium, while SNA supports each *Fusarium* species in developing its species-specific macro- and microconidia. *Fusarium* isolates were identified to species level using light microscopy (Jenaval, Carl Zeiss, Jena, Germany), and the identification was mainly based on morphological characteristics described in details by Leslie and Summerell (2006).

### Culture-independent method (qPCR approach)

For further analyses by qPCR, remaining grass and wheat ear samples were dried (60 °C for at least 48 h) and ground using a vibrating cup mill RS200 (Retsch, Haan, Germany): 1300 rpm for 1.5 min for grasses and 1000 rpm for 45 s for wheat ears. Afterwards, the grounded material (250 mg for grasses and 50 mg for wheat ears) was mixed and used for genomic DNA extraction according to the customized standard protocols of the following DNA extraction kits: NucleoSpin® Soil Kit (MACHEREY–NAGEL GmbH & Co. KG, Düren, Germany) for grasses and DNeasy Plant Mini kit (QIAGEN GmbH, Hilden, Germany) for wheat ears.

The lyses for the grass samples were adapted to plant material, and samples were centrifuged by 13,000 rpm instead of 11,000 rpm. The quantification of DNA gene copy numbers of *Fusarium* by a qPCR approach with genus-specific primers was described in detail by (Müller et al. 2018). The QuantStudio™ 12 K Flex Real-Time PCR System (Thermo Fisher Scientific Inc., Waltham, MA, USA) and the software “QuantStudio™ 12 K Flex Software v1.x” (Thermo Fisher Scientific Inc., Waltham, MA, USA) was used for the qPCR assay. Quantification of *Fusarium* gcn/gDM was performed using the HOT FIREPol® Probe GC qPCR Mix (Solis Biodyne, Tartu, Estonia). The standard curves were generated by using DNA of *F. graminearum* strain name “Fg486”. Reactions were carried out under the following thermal conditions: 95 °C for 10 min (hold stage) and 45 cycles of 95 °C for 15 s and 67 °C for 1.5 s (PCR stage). The fungal strains used for the preparation of the standard curves were stored in a culture collection of fungi of the working group “Fungal Interactions” at the ZALF in Müncheberg. All measurements were done in duplicate and qPCR assays contained negative controls. The genome copy numbers were expressed in FUS_gcn/g DM for *Fusarium* fungi.

### Analyses of deoxynivalenol and zearalenone

For the analyses of DON and ZEN, harvested and threshed samples were dried at 60 °C for at least 48 h and ground in an ultra-centrifugal mill with vibratory feeder (ZM 200, Retsch Haan Germany). The *Fusarium* toxins DON and ZEN were extracted as described in detail previously by Müller et al. (2018) and analyzed on a HPLC system consisted of an Ultimate 3000 unit (Thermo Fisher Scientific GmbH, Dreieich, Germany) combined with a diode array detector (DAD3000) and a fluorescence detector (FLD 3400RS). DON was separated on a Synergie Polar RP 100A column (2.5-μm particle size, 130 × 3 mm i.d., Phenomenex LTD, Aschaffenburg, Germany). Methanol:water (25:75, v/v) was used as eluent with a flow rate of 0.65 ml/min at 30 °C. The analyte was detected by measuring the UV-absorbance at 220 nm and 280 nm simultaneously.

ZEN was analyzed after separation on a Lichrospher 100 RP18 column (5-μm particle size, 150 × 4.6 mm i.d., VDS OptiLab Berlin, Germany) with methanol:3 mM phosphoric acid (65:35, v/v) as eluent with a flow rate of 0.65 ml/min at 25 °C. ZEN concentration was determined by fluorescence detection (extinction: 274 nm, emission: 456 nm). Mycotoxin standard substances were obtained from Romer Labs Diagnostic GmbH (Tulln, Austria). Each analysis was performed in duplicate. All toxin concentrations were calculated on the dry matter (DM) of the substrate (ng/g DM), and the toxin detection limits in the grains were 30 ng DON and 2 ng ZEN per gram of substrate DM.

### Statistics

All statistical tests were performed using SPSS (IBM SPSS Statistics V 22.0). For the visualization of the gcn/gDM of fungal abundances as boxplots, a logarithmic transformation LOG (x + 1) was applied to the data of the culture-independent method (qPCR approach). Via a Kolmogorov–Smirnov test, the abundance data of *Fusarium* (cfu/gFM and gcn/gDM) was tested for normal distribution. The differences in fungal quantities between the different *Fusarium* species (gcn/gDM), and the differences between the mycotoxin accumulations (ng/g) were compared by Kruskal–Wallis test and Mann–Whitney *U* test. In all figures, a/b indicate significant differences between parameters investigated (*p* values < 0.05).

## Results

### Microclimate

Except for air temperature, we were able to detected differences regarding the microclimatic factors on the irrigated and the non-irrigated field side. Especially in June, but also in July, we found a higher soil moisture, a higher air humidity and a higher leaf wetness on the field side with irrigation (Fig. S1).

### Inoculated grass stripe with *F. graminearum*, *F. sporotrichioides*, and *F. culmorum* in May and June

**Fig. 1:**
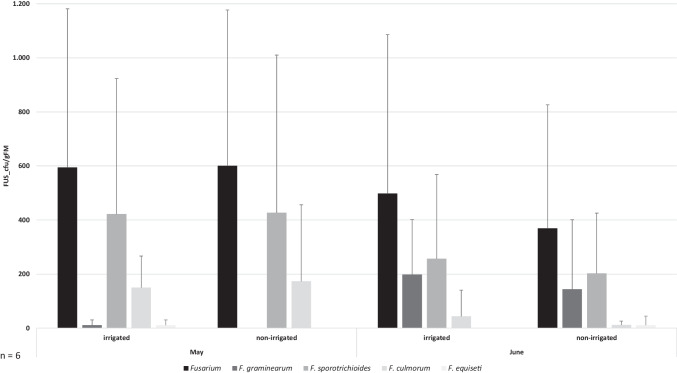
Mean values of the colony forming units of total *Fusarium* fungi (FUS_cfu/gFM), *F. graminearum* (GRAM_cfu/gFM), *F. sporotrichioides* (SPORO_cfu/gFM), *F. culmorum* (CULM_cfu/gFM), and *F. equiseti* (EQUI_cfu/gFM) on grass samples in May and June, both on the irrigated and the non-irrigated field side. The mean values are plotted with their standard deviation of the mean

The *Fusarium* abundance (FUS_cfu/gFM) determined in the two sampling months decreased from May to June (Fig. 1): the mean total abundance of *Fusarium* on the grass stripe in May was 598 FUS_cfu/gFM, compared to 434 FUS_cfu/gFM in June. The abundance of *Fusarium* was 1.4-fold lower in the second month. Regarding the irrigation, the measured abundances of the total *Fusarium* abundance in May were almost even. In June, the abundance of *Fusarium* on the irrigated field side was 1.3-fold higher compared to the field side with no irrigation. In both months, *F. sporotrichioides* was the predominant species on the grass samples investigated. The abundance of *F. sporotrichioides* (SPORO_cfu/gDM) was higher in May and decreased until June, while there was no difference associated to the irrigation detected: the abundance of *F. sporotrichioides* was almost even on both sides. Also, the abundance of *F. culmorum* decreased from May to June. In May, the abundance *F. culmorum* on the irrigated field side was even to the non-irrigated field side, and in June, the abundance on the irrigated side was 3.7-fold higher.

In contrast to the other *Fusarium* species analyzed, the abundance of *F. graminearum* (GRAM_cfu/gFM) increased from May until June. We determined 16 × more GRAM_cfu/gFM in June compared to May. In May, *F. graminearum* was only isolated in low abundances on the irrigated field side. Also in June, more *F. graminearum* (1.4-fold) was detected on the field side with irrigation, but further on the non-irrigated side as well. In May, *F. culmorum* was the second most isolated *Fusarium* species of the grass samples, while in June, it was *F. graminearum*. The abundance of *F. sporotrichioides* in May was 39-fold higher than the abundance of *F. graminearum*, while in June, the difference was only 26%.

We also isolated *F. equiseti* from the grass stripe, even though this species, compared to the others named above, was not inoculated. We found low abundances of *F. equiseti* (11 EQUI_cfu/gFM) both in May as well as in June, independently from the irrigation.

### Gene copy numbers of Fusarium spp. on wheat ears on the irrigated and the non-irrigated field side and along the transect

**Fig. 2:**
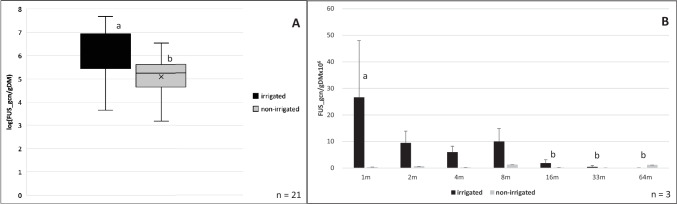
**A** Abundances of *Fusarium* fungi expressed as LOG (x + 1) (gene copy numbers) of *Fusarium* (FUS_gcn/gDM) on wheat ears on the irrigated and the non-irrigated field side in July 2021. The midline of the boxplots represents the median; the x the mean value; the upper and lower limits of the boxes the third and first quartile. a/b indicates a significant difference between parameters investigated (*p values < 0.05), **B** Mean values of the gene copy numbers of *Fusarium* (FUS_gcn/gDM) along a transect from 1 to 64 m into a wheat field on the irrigated and the non-irrigated field side. The mean values are plotted with their standard deviation of the mean. a/b indicate significant differences (*p < 0.05)

Regarding *Fusarium*, the abundance of the FUS_gcn/gDM on the irrigated field side was significantly higher compared to the non-irrigated side (Fig. 2A).

However, concerning the transect, high abundances up to 26,000,000 FUS_gcn/gDM were found at the first sampling point at 1 m next to the infected grass stripe (Fig. 2B). In 4 m distance, there is still a FUS_gcn/gDM of 6,000,000 to be found. These high *Fusarium* abundances only occurred on the irrigated field side. On the non-irrigated field side, the abundance of *Fusarium* differs from 1,250,000 to 18,300 gcn/gDM. The highest abundances on the non-irrigated field side were measured at 8 m and 64 m, the lowest at 33 m, but the differences in the abundance were not statistically significant.

Concerning *Fusarium*, there is a gradient from the grass stripe along the transect to be found on the irrigated field side. Significant differences were detected starting at 16 m away from the infected grass stripe, compared to the sampling point directly next to the stripe. From 1 to 4 m the FUS_gcn/gDM decreased, while at 8 m the abundance increased again up to the level of 2 m. In contrast, no significance along the transect could be identified on the non-irrigated field side.

### Diversity of different *Fusarium* species determined on wheat kernels in July 2021 on the irrigated and the non-irrigated field side and along the transect

Concerning the different *Fusarium* species isolated from the wheat field in July, *F. graminearum* was the dominant species on the irrigated field side (Table 1). The latter accounted for 77% of the whole *Fusarium* fungi isolated from the wheat kernels. This abundance is significantly different to the abundances of *F. sporotrichioides* (7%) and *F. culmorum* (1%), among the other detected species. Besides the three inoculated *Fusarium* species, we identified four additional *Fusarium* species on the field with irrigation: *F. equiseti*, *F. cerealis*, *F. avenaceum*, and *F. poae*. *F. equiseti* accounted for 5% of the total *Fusarium* abundance and was already isolated from the grass stripe in May and June. *F. cerealis* and *F. avenaceum* were detected for the first time during the field study and were both found in abundances of 3%. *F. poae*, also initially found in July on wheat ears, accounted for 1% of the total *Fusarium* abundance.

**Table 1:** Species composition (%) of *Fusarium* (inoculated species and other isolated species) on wheat ears in July on the irrigated and the non-irrigated field side with Shannon- and Evenness-Index. All sampling points from each field side pooled together (*n* = 48)

*Fusarium* species (%)	Irrigated field side	Non-irrigated field side
*F. graminearum*	77	20
*F. sporotrichioides*	7	17
*F. culmorum*	1	36
*F. equiseti*	5	17
*F. poae*	1	-
*F. cerealis*	1	8
*F. avenaceum*	1	2
*F. spec*	1	-
Shannon index	0.75	1.67
Evenness index	0.39	0.86

In total, seven different *Fusarium* species in different abundances were determined on the irrigated field side from wheat kernels in July, during the ripening of ears.

On the non-irrigated field side, the species composition of *Fusarium* was more balanced. *F. culmorum*, the least isolated species on the irrigated field side, was the dominant species on the non-irrigated field side and accounted for 36% of the total *Fusarium* abundance. *F. graminearum* (20%) and *F. sporotrichioides* (17%), both inoculated species together with *F. culmorum*, were also frequently detected on the non-irrigated field side. Again, *F. equiseti*, which was not inoculated at the beginning of the field experiment, was found in the same high amount as *F. sporotrichioides*. This makes *F. equiseti*, together with *F. sporotrichioides*, the third most isolated *Fusarium* species on wheat ears on the field side without irrigation. Furthermore, just like on the irrigated side, *F. cerealis* (8%), even in higher abundances, and *F. avenaceum* (2%) were isolated. We detected three additional *Fusarium* species on the non-irrigated field side. *F. poae* was only isolated from the irrigated field side.

**Fig. 3:**
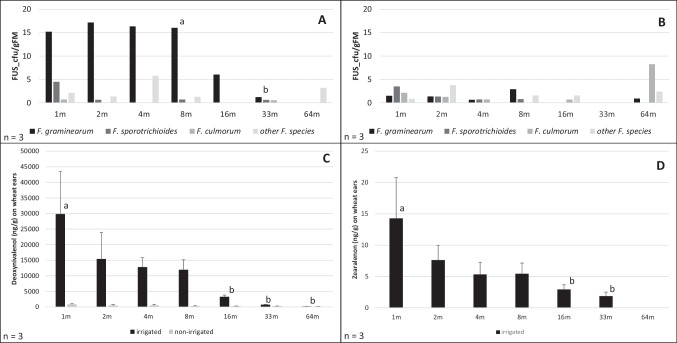
**A** Mean values of the cfu/gFM of *F. graminearum* (GRAM_cfu/gFM), *F. sporotrichioides* (SPORO_cfu/gFM), *F. culmorum* (CULM_cfu/gFM) and other *Fusarium* species (OTHER_cfu/gFM) along a transect from 1 to 64 m into a wheat field on the irrigated field side. a/b indicate significant differences (*p < 0.05), **B** Mean values of the cfu/gFM of *F. graminearum* (GRAM_cfu/gFM), *F. sporotrichioides* (SPORO_cfu/gFM), *F. culmorum* (CULM_cfu/gFM) and other *Fusarium* species (OTHER_cfu/gFM) along a transect from 1 to 64 m into a wheat field on the non-irrigated field side. **C** Mean values of the DON concentration in ng/g on wheat ears from the irrigated and the non-irrigated field side along a transect from 1 to 64 m into a wheat field. The mean values are plotted with their standard deviation of the mean. a/b indicate significant differences (*p < 0.05), **D** Mean values of the ZEN concentration in ng/g on wheat ears from the irrigated field side along a transect from 1 to 64 m into a wheat field. The mean values are plotted with their standard deviation of the mean. Asterisk brackets indicate significant differences (*p < 0.05) ZEN was under the toxin detection limit of 2 ng/g at sampling point 64 m on the irrigated field side and on all sampling points on the non-irrigated field side

On the irrigated field side, *F. graminearum*, predominant species on this field side, was detected in similar high abundances from 1 m up to 8 m along the transect (Fig. 3A). Between 8 and 33 m away from the inoculated grass stripe, there was a decrease in the abundance of *F. graminearum* to be found, just as between 16 and 33 m. But only between ears at 8 m and 33 m, the difference was significant. No isolates of *F. graminearum* were determined from 64 m away from the grass stripe, also none of *F. sporotrichioides* or *F. culmorum*.

At 1 m, directly next to the grass stripe, *F. sporotrichioides* was the second most detected species, but with abundances 3.4-fold lower than the abundance of *F. graminearum* on wheat ears. At 2 m, 8 m, and 11 m, we also isolated *F. sporotrichioides*, but only in low abundances (0.61–0.71 SPORO_cfu/gFM). *F. culmorum* was only determined at two sampling points: 1 m and 33 m. Compared to *F. graminearum*, the abundance of *F. culmorum* at 1 m was 21.4-fold lower, 6.3-fold lower compared to *F. sporotrichioides*. We also isolated four additionally *Fusarium* species, which were not inoculated at the grass stripe in November 2020. These species were detected at all sampling points from 1 m up to 8 m and again at 64 m. At 1 m, the abundance of not inoculated *Fusarium* species was threefold higher compared to the abundance of *F. culmorum*. Regarding the irrigated field side, none of the primary inoculated *Fusarium* species were detected at the last sampling point, 64 m away from the infected grass stripe.

On the non-irrigated field side, the abundances of all three inoculated *Fusarium* species are decreasing from 1 m up to 4 m along the transects (Fig. 3B). *F. sporotrichioides* was the predominant detected species isolated from wheat ears at 1 m, next to the artificial infected grass stripe, followed by *F. culmorum*. The abundance of *F. graminearum* was 2.4-fold lower compared to *F. sporotrichioides*. At 2 m and 4 m, the abundances of the three inoculated species were even. *F. sporotrichioides* was isolated up to 8 m into the field without irrigation. *F. culmorum* was determined from 1 to 4 m in decreasing abundances. Concerning additionally detected *Fusarium* species (Fig. 3A/B), we isolated those at all sampling points except 4 m and 33 m. At 2 m, the other *Fusarium* species were dominant, and also at 8 m, 6 m, and 64 m, additionally occurring *Fusarium* species were found in higher abundances than some of the inoculated species: at 8 m, the abundance of *F. sporotrichioides* was twofold lower compared to the other species, at 16 m, we determined the same for *F. culmorum*. Additionally occurring species were the second most detected *Fusarium* species at 64 m, but found in 3.4-fold lower abundances than *F. culmorum*. All species were isolated in low abundances: no statistically significant differences between the cfu/gDM of the different species were detected.

### Analyses of DON and ZEN concentration along the transects on the irrigated and the non-irrigated field side

The highest levels of DON in wheat kernels were detected on the irrigated field side. Up to 30,000 ng/g DON were analyzed via HPLC at the first sampling point directly next to the infected grass stripe (Fig. 3C). Along the transect into the wheat field, the DON concentration decreased until 110 ng/g at 64 m. There were significant differences in the mycotoxin concentration in wheat kernels on the irrigated field side at 16 m, 33 m, and 64 m, compared to the sampling point next to the infected grass stripe (1 m). The highest decrease in DON concentration was measured between 8 and 16 m: 72.5% less DON at 16 m. At the last two sampling points (33 m and 64 m), only a low concentration of DON of 718 ng/g and 110 ng/g were detected via HPLC, especially compared to the concentration at sampling point 1 m.

Concerning the non-irrigated field side, the DON concentration along the transect is lower compared to the irrigated side. A trending decrease of the DON concentrations from 790 to 124 ng/g was measured, but none of the differences between the sampling points along the transect were statistically significant.

Regarding ZEN, a mycotoxin contamination up to 14 ng/g at 1 m, next to the infected grass stripe, was measured. At the last sampling point at 64 m, no ZEN accumulation in a recognizable amount was detected (Fig. 3D). From 1 to 4 m the ZEN concentration decreased, while it is even at 4 m and 8 m. After 8 m, it decreased again until the end of the transect. The measured differences were significant from 1 to 16 m and also 33 m. The ZEN accumulation on the irrigated field side never exceeded the toxin regulations set by the EU, and no ZEN contamination was found on the non-irrigated field side.

Regarding the yield, no statistically significant differences were detected: the yield was almost even at the irrigated and the non-irrigated field side.

## Discussion

In the present study, an inoculated grass stripe was analyzed as reservoir for an infection with *Fusarium* spp. for an adjacent wheat field. The grass stripe was highly infected with three different species of the genera *Fusarium*: *F. graminearum*, *F. culmorum*, and *F. sporotrichioides*. Furthermore, the distribution of *Fusarium* along a transect into to field, the *Fusarium* species composition on the wheat ears and the mycotoxin contamination of the wheat kernels were determined. We could clearly show that infection has spread from the grass stripe up to 33 m into the wheat field, as evidenced by the increased abundance of *Fusarium* fungi as well as mycotoxin accumulation in the wheat ears near the grass stripe. This effect was remarkably enhanced by altered microclimatic conditions (humidity and temperature) that prevailed on one side of the field as a result of the irrigation.

Gerling et al. (2022) measured the *Fusarium* abundance of different weed species at the edges of semi-natural landscape elements in arable lands, finding out that grasses are a more attractive habitat for different *Fusarium* species than herbaceous plants. Both, the fungal abundance and the species diversity was highest on grass samples. In the field experiment shown here, we were able to create a strong infection of grass stripe due to soil inoculation up to 600 FUS_cfu/gDM. This seems to confirm the findings of the just cited study: grasses are suitable hosts for different species of *Fusarium* fungi. It is assumed that different *Fusarium* species and their community composition on both grasses and wheat are strongly determined by abiotic factors such as soil and air humidity and temperature as well as by their own competitive strength, aggressiveness and spore dispersal. It was general consent that *F. graminearum* was limited to regions with warm temperature (28 °C) and with moderate to high rainfall during anthesis (Parry et al. 1993; Backhouse and Burgess 2002; Xu and Nicholson 2009) and therefore was the most common causal agent of FHB in the USA, in Canada, Australia, and parts of central Europe (Goswami and Kistler 2004; Osborne and Stein 2007). In cooler, maritime regions like the UK and northern Europe, *F. culmorum* was prevalent (Parry et al. 1993; Jennings et al. 2004; Gilbert et al. 2013). Due to rising temperatures, *F. graminearum* incidences increased also in regions where *Fusarium culmorum* was the main causal agent of FHB (Waalwijk et al. 2003; Xu et al. 2005). Different studies confirm the increase of *F. graminearum* and the decrease of *F. culmorum* over the last two decades in the UK (Jennings et al. 2004), in Netherlands (Waalwijk et al. 2003), in Denmark (Nielsen et al. 2011), in Poland (Stępień and Chełkowski 2010) and in Germany (Miedaner et al. 2008), which may reflect the climatic effects on these species. The results of the present study regarding the abundance of *F. graminearum* and *F. culmorum* in the grass stripe showed a similar pattern but in the small-scale dimensions of our field experiment. Both species were inoculated in the grass stripe. In May, the month with lower temperature, higher abundances of *F. culmorum* were isolated from the grasses, while no isolates of *F. graminearum* were found. In June, the temperature raised and also the *F. graminearum* incidence increased, while the incidence of *F. culmorum* decreased. *F. sporotrichioides* also used as an inoculum, was the predominant isolated species in both months on the grass stripe, followed by *F. culmorum* in May and *F. graminearum* in June. This leads to the assumption that *Fusarium* species which are better adapted to rising temperatures may become more prevalent in the future and that the high infestation of *F. graminearum* indicates a better adaption to changing environmental conditions than *F. culmorum* (Panwar et al. 2016). However, this assumption seems to be confirmed for grasses as non-crop host plants colonizing as alternate and alternative hosts. *F. sporotrichioides* is a causal agent of FHB, but is not as often isolated as *F. graminearum* or *F. culmorum* (Salas et al. 1999; Lenc et al. 2015), but on grasses, it seems to be competitive, even against *F. graminearum*. *F. sporotrichioides* is mainly to be found in temperate regions, but has a temperature range from approximately − 2 to 35 °C (Thrane 2014). The infection of inflorescences of host plants through this species occurs from 10 to 40 °C (Nazari et al. 2014). Gerling et al. (2022) analyzed the abundance of different *Fusarium* species on arable weeds, including grasses, at the edges of kettle holes in two consecutive autumn/winter seasons with the result that *F. sporotrichioides* was the dominant isolated species in both seasons. In the present study, *F. sporotrichioides* was detected on grasses and wheat ears, but in lower abundances than *F. graminearum* (irrigated field side) and *F. culmorum* (non-irrigated field side). The competitiveness of *F. sporotrichioides* against other *Fusarium* species seems to be better on grass-hosts, compared to wheat plants.

*Fusarium* fungi colonized the wheat plants in high frequencies and abundances and produced remarkable mycotoxin amounts in the field, especially at the sampling points near the infected grass stripe. This result clearly shows that the infected grass stripe influenced the fungal abundance of *Fusarium* in the wheat field and the mycotoxin accumulation in the wheat kernels at harvest time. The *Fusarium* species composition on the wheat ears was strongly influenced by the grass stripe growing next to the field and the *Fusarium* spores transported by wind and plant-to-plant contact from there. The previously inoculated *Fusarium* species account for 85% (irrigated field side) and 73% (non-irrigated field side) of the total *Fusarium* abundance, but in different abundances for the species. This result clearly shows the immigration of the inoculated *Fusarium* species from the grass stripe into the field, which makes a *Fusarium* infected grass stripe next to a wheat field a reservoir for fungal infections. This pattern was determined on both field sides but seems to be strongly promoted by the irrigation. The species composition of *Fusarium* depends on several factors, especially on the precipitation and the temperature at flowering stage (Bottalico and Perrone 2002; Covarelli et al. 2015). Therefore, due to climate change, shifts in the composition of *Fusarium* species on cereal grains in Northern Europe are expected (Parikka et al. 2012). Regarding further frequently detected *Fusarium* species in our study, *F. equiseti* is mainly known as a saprophytic species, which can also be a causal agent for FHB, but not as aggressive as *F. graminearum* or *F. culmorum* (Langseth et al. 1999). Desai et al. (2020) suggested that high intense rainfall events could promote the saprophytic living *Fusarium* species. In the present study, this species was more frequent detected on wheat kernels of the non-irrigated field side. *F. equiseti* was also isolated from the irrigated field side, but in lower abundances. Maybe the saprophytic *F. equiseti* was not able to compete with the more aggressive *F. graminearum* species, which was the dominant species on the irrigated field side.

A comparison with other studies that have investigated the extent to which the effects of edge structures or transition zones affect the neighboring agricultural field is difficult because there are only a few of them.

A study of Raatz et al. (2019) shows that yield decreases next to semi-natural landscape elements up to a distance of 11.3 m from kettle holes and up to a distance of 17.8 m from hedgerows and forest borders. The authors named shade, soil compaction through the use of machinery and also a reduced use of chemical preparations close to water bodies as the main reasons. In a second study, Raatz et al. (2021) analyzed different pests and pathogens regarding their ability to decrease yield from semi-natural landscape elements into wheat fields, but did not find any evidence of yield reduction due to an infection with pathogenic fungi, although the infection rate of wheat leaves next to kettle holes was higher compared to the mid field. This is in agreement with our results: The present study shows no significant differences in yield between the irrigated and non-irrigated field sides, although the former was much more infested with filamentous fungi of the genera *Fusarium*. Raatz et al. (2021), however, only analyzed the yield quantity, no qualitative parameters. But an infection of wheat with, e.g., FHB can cause both, quantitative yield losses and qualitative changes of nutrients in wheat grains including an accumulation of toxic substances (Martínez et al. 2020): Quality losses in wheat are primarily caused by the contamination of the harvested crop with mycotoxins. If the contamination exceeds the limits set by the EU, the crop may no longer could be sold, fed, or processed and must be destroyed. The limits for DON and ZEN in unprocessed cereals are set by the European Commission regulation (EU) at 1250 ng/g for DON and 100 ng/g for ZEN (EC 2006).

In our experiment, we investigated both, the yield, and the mycotoxin contamination of grains at the harvest. The results show a significantly increased toxin accumulation on the field side which was irrigated. This was determined for both the analyzed mycotoxins, but mainly for DON. The limits for DON were exceeded with a maximum value of 29.000 ng/g on the irrigated field side at the sampling point directly next to the grass stripe. We determined high DON contaminations of the grains up to 16 m into the field. Only at a distance of 33 m from the grass stripe did the DON concentration decline to values below the EU limit (718 ng/g) and at the end of the transect (64 m away from the grass stripe), we analyzed only a low concentration of DON (110 ng/g). On the dryer field side, the limits for DON were not exceeded, same for the limits of ZEN on both field sides, independent from the irrigation. But also, for DON concentration of the non-irrigated field side and for ZEN, there was a decrease to be detected: the highest levels of mycotoxin contamination were analyzed at the sampling points right next to the infected grass stripe.

On both sides of the field, the inoculated *Fusarium* species were detected, but in different frequencies and abundances regarding the irrigation. A higher *Fusarium* load on wheat ears was found at the sampling points near to the grass stripe compared to the middle and end of the transect (at the opposite side of the field). Therefore, it can be assumed that the *Fusarium* species inoculated in the grass strip have spread into the wheat field, even if the final confirmation that they are the same strains is lacking. The results show clearly that grasses at the edges of fields can act as reservoirs for *Fusarium* and spread the infection on the wheat plants growing next to them, especially when the grasses are highly infected. Gerling et al. (2022) already show that concerning arable weeds, grasses are the most *Fusarium*-infected weed species in Brandenburg. The influence of the grass stripe on the infection of the wheat plants is enhanced by temperature and moisture conditions, especially at the flowering stage (Leplat et al. 2013; Martínez et al. 2020). This effect was most notable for *F. graminearum*.

Grasses seem to be an attractive alternative host for different *Fusarium* species, especially when the main host is absent. Therefore, grasses as non-crop host plants and reservoirs for fungal pathogens will become more important in the next decades and need to be considered in the FHB management. Due to increasing temperatures, *F. graminearum* may become the dominant species in further parts of (northern-) Europe. Knowledge about a possible shift in the FHB species profile is essential, first of all since *F. graminearum* is considered the most important trigger of FHB and as the most aggressive DON producer. According to our study, *F. graminearum*, under beneficial microclimatic conditions, has the potential to spread from a source of infection up to 33 m into a wheat field. As the global food demand is expected to double by 2050 (Godfray et al. 2010), it is more important than ever to ensure the food security of wheat due to an improved FHB management. Knowledge about changes in the intensity of FHB epidemics caused by changing climatic conditions is key for an effective management of FHB in the future. By trying to increase the biodiversity in arable lands, the number of semi-natural landscape elements (e.g., hedgerows, small forests, flower stripes) will increase and along with this also the amount of transition zones between these two habitats.

Our results show that grasses at the edges of wheat fields increase the risk of an infection with FHB for the adjacent wheat field, mainly for the field parts directly next to the grasses. Because of this, we recommend to take grasses into account as a source of infection for FHB, especially after rain events while flowering or when the field is irrigated. Future work is needed to analyze the influence of grasses on the abundance and distribution of more different *Fusarium* species into wheat fields, especially those belonging to the FHB complex.

## Supplementary Information

Below is the link to the electronic supplementary material.

Supplementary file1 (PDF 243 KB)

## Data Availability

The datasets generated during and/or analyzed during the current study are available from the corresponding author on reasonable request.

## Abbreviations

cfu: Colony forming units
gcn: Gene copy numbers
FUS_cfu/gFM: *Fusarium* Colony forming units per gram fresh matter
GRAM_cfu/gFM: *F. graminearum* Colony forming units per gram fresh matter
CULM_cfu/gFM: *F. culmorum* Colony forming units per gram fresh matter
SPORO_cfu/gFM: *F. sporotrichioides* Colony forming units per gram fresh matter
EQUI_cfu/gFM: *F. equiseti* Colony forming units per gram fresh matter
FUS_gcn/gDM: *Fusarium* Gene copy numbers per gram dry matter
FHB: Fusarium head blight
DON: Deoxynivalenol
ZEN: Zearalenone
ZALF: Leibniz Center for Agricultural Landscape Research

## References

[CR1] Anonymous (2022) Federal Plant Variety Office (Bundessortenamt). Beschreibende Sortenliste 2022 –Getreide, Mais, Öl- und Faserpflanzen, Leguminosen, Rüben, Zwischenfrüchte. ISSN 2190–6130

[CR2] Backhouse D, Abubakar AA, Burgess LW, Dennis JI, Hollaway GJ, Wildermuth GB, Wallwork H, Henry FJ (2004). Survey of *Fusarium* species associated with crown rot of wheat and barley in eastern Australia. Australas Plant Pathol.

[CR3] Backhouse D, Burgess LW (2002). Climatic analysis of the distribution of *Fusarium graminearum*, *F. pseudograminearum* and *F. culmorum* on cereals in Australia. Australas Plant Pathol.

[CR4] Benton TG, Vickery JA, Wilson JD (2003). Farmland biodiversity: is habitat heterogeneity the key?. Trends Ecol Evol.

[CR5] Bottalico A (1998) *Fusarium* disease of cereals: species complex and related mycotoxin Profiles, in Europe. J Plant Pathol 80:85–103. https://www.jstor.org/stable/41997909. Accessed 2 May 2022

[CR6] Bottalico A, Perrone G (2002). Toxigenic *Fusarium* species and mycotoxins associated with head blight in small-grain cereals in Europe. Eur J Plant Pathol.

[CR7] Champeil A, Fourbet JF, Doré T, Rossignol L (2004). Influence of cropping system on Fusarium head blight and mycotoxin levels in winter wheat. Crop Prot.

[CR8] Chhaya RS, O’Brien J, Cummins E (2021). Feed to fork risk assessment of mycotoxins under climate change influences - recent developments. Trends Food Sci Technol.

[CR9] Classen AT, Sundqvist MK, Henning JA, Newman GS, Moore JAM (2005). Direct and indirect effects of climate change on soil microbial and soil microbial-plant interactions: What lies ahead?. Ecosphere.

[CR10] Covarelli L, Beccari G, Prodi A, Generotti S, Etruschi F, Juan C (2015). *Fusarium* species, chemotype characterisation and trichothecene contamination of durum and soft wheat in an area of central Italy. J Sci Food Agric.

[CR11] Desai S, Dubey SC, Prasad RD (2020). Impacts of climate change on *Fusarium* species vis-à-vis adaptation strategies. Indian Phytopathol.

[CR12] Dill-Macky R, Jones RK (2000). The effect of previous crop residues and tillage on Fusarium head blight of wheat. Plant Dis.

[CR13] Dong F, Xu J, Zhang X, Wang S, Xing Y (2020). Gramineous weeds near paddy fields are alternative hosts for the *Fusarium graminearum* species complex that causes fusarium head blight in rice. Plant Pathol.

[CR14] Drakopoulos D, Kägi A, Six J, Zorn A, Wettstein FE (2021). The agronomic and economic viability of innovative cropping systems to reduce *Fusarium* head blight and related mycotoxins in wheat. Agric Syst.

[CR15] EC – European Commission (2006) Commission regulation (EC) No 1881/2006 of 19 December 2006 Setting maximum levels for certain contaminants in foodstuffs. Off J Eur Union L. 364:5–24 Last consolidated version available from: https://eur-lex.europa.eu/legal-content/DE/AUTO/?uri=CELEX:02006R1881-20180319. Accessed 12 May 2022

[CR16] Figueroa M, Hammond-Kosack KE, Solomon PS (2018). A review of wheat diseases—a field perspective. Mol Plant Pathol.

[CR17] Fulcher MR, Garcia JP, Damann KCM, Bergstrom GC (2019). A) Variable interactions between non-cereal grasses and *Fusarium graminearum*. Can J Plant Pathol.

[CR18] Fulcher MR, Winans JB, Quan M, Oladipo ED, Bergstrom GC (2019). Population genetics of *Fusarium graminearum* at the interface of wheat and wild grass communities in New York. Phytopathology.

[CR19] Gerling M, Pätzig M, Hempel L, Büttner C, Müller MEH (2022). Arable weeds at the edges of kettle holes as overwintering habitat for phytopathogenic fungi. Agronomy.

[CR20] Gilbert HJ, Knox JP, Boraston AB (2013). Advances in understanding the molecular basis of plant cell wall polysaccharide recognition by carbohydrate-binding modules. Curr Opin Struct Biol.

[CR21] Godfray HCJ, Beddington JR, Crute IR, Haddad L, Lawrence D, Muir JF, Pretty J, Robinson S, Thomas SM, Toulmin C (2010). Food security: The challenge of feeding 9 billion people. Science.

[CR22] Góral T, Łukanowski A, Małuszyńska E, Stuper-Szablewska K, Buśko M, Perkowski J (2019) Performance of winter wheat cultivars grown organically and conventionally with focus on fusarium head blight and *fusarium* trichothecene toxins. Microorganisms 7(10). 10.3390/MICROORGANISMS7100439 10.3390/microorganisms7100439 PMC6843174 31614527

[CR23] Goswami RS, Kistler C (2004). Heading for disaster: *Fusarium graminearum* on cereal crops. Mol Plant Pathol.

[CR24] Hansen B, Fjelsted Alrøe H, Steen Kristensen E (2001). Approaches to assess the environmental impact of organic farming with particular regard to Denmark. Agric Ecosyst Environ.

[CR25] Heitmann N, Glemnitz M, Lentzsch P, Platen R, Müller MEH (2021). Quantifying the role of ground beetles for the dispersal of *fusarium* and *alternaria* fungi in agricultural landscapes. J Fungi.

[CR26] Hoffmann A, Funk R, Müller MEH (2021). Blowin’ in the wind: Wind dispersal ability of phytopathogenic *fusarium* in a wind tunnel experiment. Atmosphere.

[CR27] Jennings P, Coates ME, Walsh K, Turner JA, Nicholson P (2004). Determination of deoxynivalenol- and nivalenol-producing chemotypes of *Fusarium graminearum* isolated from wheat crops in England and Wales. Plant Pathol.

[CR28] Kelly AC, Clear RM, O’Donnell K, McCormick S, Turkington TK, Tekauz A, Gilbert J, Kistler HC, Busman M, Ward TJ (2015). Diversity of Fusarium head blight populations and trichothecene toxin types reveals regional differences in pathogen composition and temporal dynamics. Fungal Genet Biol.

[CR29] Keller MD, Bergstrom GC, Shields EJ (2014). The aerobiology of *Fusarium graminearum*. Aerobiologia.

[CR30] Kumar S, Bhowmick MK, Ray P (2021). Weeds as alternate and alternative hosts of crop pests. Indian J Weed Sci.

[CR31] Landschoot S, Audenaert K, Waegeman W, Pycke B, Bekaert B (2011). Connection between primary *Fusarium* inoculum on gramineous weeds, crop residues and soil samples and the final population on wheat ears in Flanders, Belgium. Crop Prot.

[CR32] Langseth W, Bernhoft A, Rundberget T, Kosiak B, Gareis M (1999). Mycotoxin production and cytotoxicity of *Fusarium* strains isolated from Norwegian cereals. Mycopathologia.

[CR33] Lenc L, Czecholiński G, Wyczling D, Turów T, Kaźmierczak A (2015). Fusarium head blight (FHB) and *Fusarium* spp. on grain of spring wheat cultivars grown in Poland. J Plant Prot Res.

[CR34] Leplat J, Friberg H, Abid M, Steinberg C (2013). Survival of *Fusarium graminearum*, the causal agent of Fusarium head blight. A Review Agron Sustain Dev.

[CR35] Leslie JF, Summerell BA (2006) The *Fusarium* laboratory manual. First Edition. Blackwell Publishing. 10.1002/9780470278376

[CR36] Liu C, Van Der Fels-Klerx HJ (2021). Quantitative modeling of climate change impacts on mycotoxins in cereals: a review. Toxins.

[CR37] Lofgren LA, Leblanc NR, Certano AK, Nachtigall J, Labine KM (2018). *Fusarium graminearum*: pathogen or endophyte of North American grasses?. New Phytol.

[CR38] Lozada-Gobilard S, Landivar Albis CM, Rupik KB, Pätzig M, Hausmann S (2021). Habitat quality and connectivity in kettle holes enhance bee diversity in agricultural landscapes. Agric Ecosyst Environ.

[CR39] Martínez M, Albuquerque LM, Arata AF, Biganzoli F, Pinto VF (2020). Effects of *Fusarium graminearum* and *Fusarium poae* on disease parameters, grain quality and mycotoxins contamination in bread wheat (Part I). J Sci Food Agric.

[CR40] Martínez M, Arata AF, Fernández MD, Stenglein SA, Dinolfo MI (2021). *Fusarium* species richness in mono- and dicotyledonous weeds and their ability to infect barley and wheat. Mycol Prog.

[CR41] Miedaner T, Cumagun CJR, Chakraborty S (2008). Population genetics of three important head blight pathogens *Fusarium graminearum*, *F. pseudograminearum* and *F. culmorum*. J Phytopathol.

[CR42] Miedaner T, Gwiazdowska D, Waśkiewicz A (2017). Editorial: management of *Fusarium* species and their mycotoxins in cereal food and feed. Front Microbiol.

[CR43] Mourelos CA, Malbrán I, Balatti PA, Ghiringhelli PD, Lori GA (2014). Gramineous and non-gramineous weed species as alternative hosts of *Fusarium graminearum*, causal agent of Fusarium head blight of wheat, in Argentina. Crop Prot.

[CR44] Müller MEH, Steier I, Köppen R, Siegel D, Proske M, Korn U, Koch M (2012). Cocultivation of phytopathogenic *Fusarium* and *Alternaria* strains affects fungal growth and mycotoxin production. J Appl Microbiol.

[CR45] Müller T, Ruppel S, Behrendt U, Lentzsch P, Müller MEH (2018). Antagonistic potential of fluorescent pseudomonads colonizing wheat heads against mycotoxin producing *Alternaria* and *Fusaria*. Front Microbiol.

[CR46] Nazari L, Pattori E, Terzi V, Morcia C, Rossi V (2014). Influence of temperature on infection, growth, and mycotoxin production by *Fusarium langsethiae* and *Fusarium sporotrichioides* in durum wheat. Food Microbiol.

[CR47] Nielsen LK, Jensen JD, Nielsen GC, Jensen JE, Spliid NH (2011). Fusarium head blight of cereals in Denmark: species complex and related mycotoxins. Phytopathology.

[CR48] Nierenberg H (1976) Untersuchungen über die Morphologische und Biologische Differenzierung in der *Fusarium*-Sektion *Liseola*. In: Mitteilungen aus der Biologischen Bundesanstalt für Land- und Forstwirtschaft Berlin-Dahlem - 169

[CR49] Oerke EC (2006). Crop losses to pests. J Agric Sci.

[CR50] Osborne LE, Stein JM (2007). Epidemiology of Fusarium head blight on small-grain cereals. Int J Food Microbiol.

[CR51] Panwar V, Aggarwal A, Kumar J, Paul S, Saharan MS (2016) Distribution dynamics of *Fusarium* spp. causing Fusarium head blight (FHB) in wheat at different geographical locations in India. South Asian J Exp Biol 6:167–177. 10.38150/sajeb.6(5).p167-177

[CR52] Parikka P, Hakala K, Tiilikkala K (2012). Expected shifts in *Fusarium* species’ composition on cereal grain in Northern Europe due to climatic change. Food Addit Contam - Part A Chem Anal Control Expo Risk Assess.

[CR53] Parry DW, Jenkinson P, Mcleod L (1993). *Fusarium* ear blight (scab) in small grain cereals-a review. Ptant Pathol.

[CR54] Pasquali M, Beyer M, Audenaert LA, K, Balmas V,  (2016). A European database of Fusarium graminearum and F. culmorum Trichothecene genotypes. Front Microbiol.

[CR55] Paul PA, El-Allaf SM, Lipps PE, Madden LV (2004). Rain splash dispersal of *Gibberella zeae* within wheat canopies in Ohio. Phytopathology.

[CR56] Postic J, Cosic J, Vrandecic K, Jurkovic D, Saleh AA, Leslie JF (2012). Diversity of *Fusarium* species isolated from weeds and plant debris in Croatia. J Phytopathol.

[CR57] Raatz L, Bacchi N, Pirhofer Walzl K, Glemnitz M, Müller MEH, Joshi J, Scherber C (2019). How much do we really lose?—Yield losses in the proximity of natural landscape elements in agricultural landscapes. Ecol Evol.

[CR58] Raatz L, Pirhofer Walzl K, Müller MEH, Scherber C, Joshi J (2021). Who is the culprit: is pest infestation responsible for crop yield losses close to semi-natural habitats?. Ecol Evol.

[CR59] Rampersad SN (2020). Pathogenomics and management of *Fusarium* diseases in plants. Pathogens.

[CR60] Rossi V, Languasco L, Pattori E, Giosuè S (2002) Dynamics of airborne *Fusarium* macroconidia in wheat fields naturally affected by head blight. J Plant Pathol 84:53–64. https://www.jstor.org/stable/41998080. Accessed 20 May 2022

[CR61] Salas B, Steffenson BJ, Casper HH, Tacke B, Prom LK, Fetch TGJ, Schwarz PB (1999). *Fusarium* species pathogenic to barley and their associated mycotoxins. Plant Dis.

[CR62] Savary S, Willocquet L, Pethybridge SJ, Esker P, Mcroberts N, Nelson A (2019). The global burden of pathogens and pests on major food crops. Nat Ecol Evol.

[CR63] Stępień Ł, Chełkowski J (2010). Fusarium head blight of wheat: pathogenic species and their mycotoxins. World Mycotoxin J.

[CR64] Suproniene S, Kadziene G, Irzykowski W, Sneideris D, Ivanauskas A (2019). Weed species within cereal crop rotations can serve as alternative hosts for *Fusarium graminearum* causing *Fusarium* head blight of wheat. Fungal Ecol.

[CR65] Thrane U (2014) *Fusarium*. In: Batt CA, Tortorello ML, editors. Encyclopedia of food microbiology. 2nd ed. Elsevier p 76–81

[CR66] Visconti A, Minervini F, Solfrizzo M, Bottalico C, Lucivero G (1992). Toxicity of some *Fusarium* section *Sporotrichiella* strains in relation to mycotoxin production. Appl Environ Microbiol.

[CR67] Vogelgsang S, Beyer M, Pasquali M, Jenny E, Musa T (2019). An eight-year survey of wheat shows distinctive effects of cropping factors on different *Fusarium* species and associated mycotoxins. Eur J Agron.

[CR68] Waalwijk C, Kastelein P, De Vries I, Kerényi Z, Van Der Lee T (2003). Major changes in *Fusarium* spp. in wheat in the Netherlands. Eur J Plant Pathol.

[CR69] Xu X, Nicholson P (2009). Community ecology of fungal pathogens causing wheat head blight. Annu Rev Phytopathol.

[CR70] Xu XM, Parry DW, Nicholson P, Thomsett MA, Simpson D (2005). Predominance and association of pathogenic fungi causing *Fusarium* ear blight in wheat in four European countries. Eur J Plant Pathol.

[CR71] Zadoks JC, Chang TT, Konzak CF (1974). A decimal code for the growth stages of cereals. Weed Res.

[CR72] Zain ME (2011). Impact of mycotoxins on humans and animals. J Saudi Chem Soc.

