# Supplementary material for: Infected grasses as inoculum for Fusarium infestation and mycotoxin accumulation in wheat with and without irrigation
Source: Mycotoxin Res. 2022 Oct 25;39(1):19–31. doi: 10.1007/s12550-022-00470-2 (PMC10156776; doi:10.1007/s12550-022-00470-2)
Supplement: Supplementary file 1 — Supplementary file1 (PDF 243 KB) [file 12550_2022_470_MOESM1_ESM.pdf]

Infected grasses as inoculum for *Fusarium* infestation and mycotoxin accumulation in wheat with and without irrigation

Marina Gerling [1,2,]*, Laura Petry [1,2], Dietmar Barkusky [1], Carmen Büttner [2] and Marina E. H. Müller [1]

[1]Leibniz Centre for Agricultural Landscape Research (ZALF), Eberswalder Str. 84, 15374 Müncheberg, Germany; mmueller@zalf.de, dbarkusky@zalf.de
[2]Albrecht Daniel Thaer-Institute, Faculty of Life Science, Department of Phytomedicine, Humboldt-Universität zu Berlin, 14195 Berlin, Germany; carmen.buettner@agrar.hu-berlin.de; https://orcid.org/0000-0002-2086-2594; laura.petry.4@gmail.com
*Correspondence: marina.gerling@zalf.de or marina.gerling@web.de; https://orcid.org/0000-0001-7039-5499; Tel.: +4915203678348

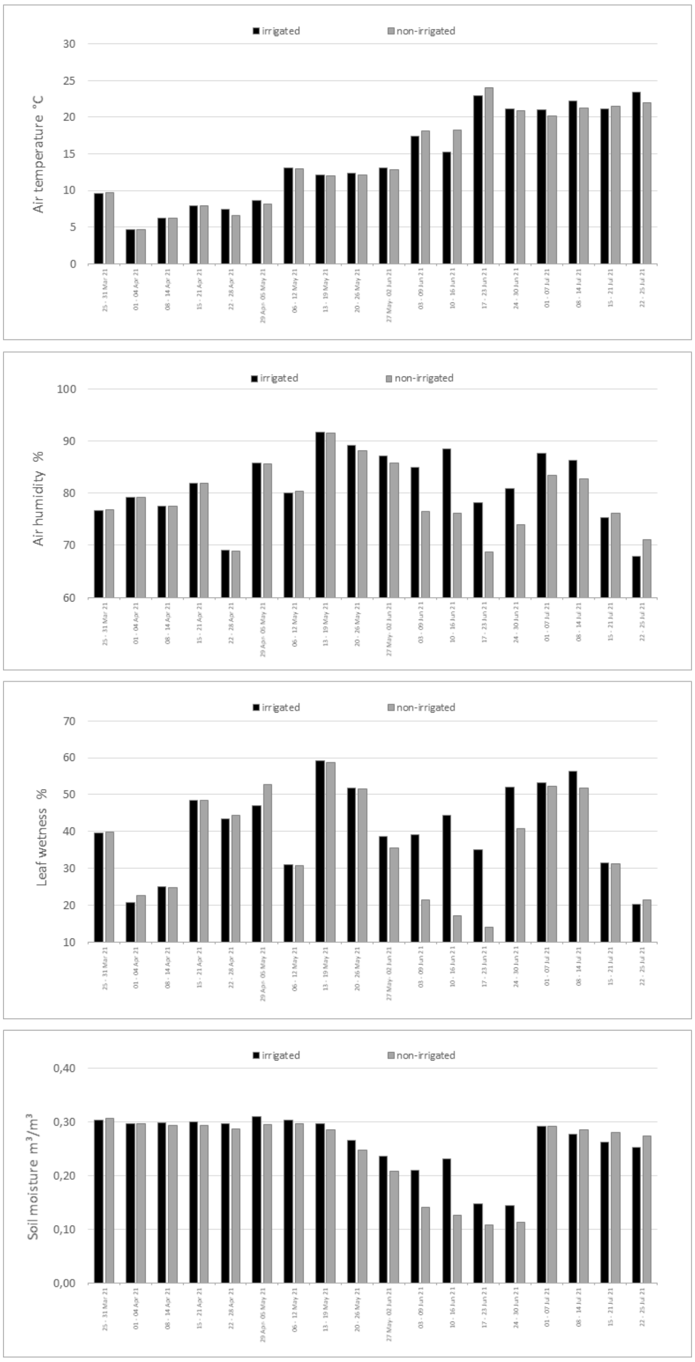

Fig. S1. Mean values of the climatic data (soil moisture in m³/m³, leaf wetness in %, air temperature in °C, air humidity in %) measured from 25th of March until the 25th of July on the irrigated and the non-irrigated field

Table S1. Detailed information on the agricultural management practices and applications.

Date	Treatment	BBCH-scale	Medium
30.09.2020	sowing	-	-
02.09.2020	fertilization	-	magnesium lime<br>(25 quintals/ha)
05.03.2021	fertilization	18	50 kg nitrogen/ha<br>12 kg sulfurl/ha
09.03.2021	fertilization	19	45 kg magnesium/ha<br>60 kg sulfur/ha
21.04.2021	fertilization	29	110 kg nitrogen/ha<br>26 kg sulfur/ha
20.10.2020	pesticide	12	Sunfire 0,25 l/ha<br>Viper Compact 0,8 l/ha
19.04.2021	pesticide	29	Sunfire 0,25 l/ha<br>A-cucel mit 1 l/ha

Table S1. Detailed information on the agricultural management practices and applications.
